# Evaluation of intravenous amoxicillin-clavulanate use in two Canadian hospitals

**DOI:** 10.1017/ash.2024.18

**Published:** 2024-02-14

**Authors:** Maggie Wong, Sangita Malhotra, Kevin Afra

**Affiliations:** 1 Department of Pharmacy, Royal Columbian Hospital, New Westminster, BC, Canada; 2 Division of Infectious Diseases, Department of Medicine, University of British Columbia, Vancouver, BC, Canada; 3 Division of Infectious Diseases, Department of Medicine, Fraser Health Authority, New Westminster, BC, Canada; 4 Division of Infectious Diseases, Department of Medicine, Fraser Health Authority, Surrey, BC, Canada

## Abstract

We describe our experience with intravenous amoxicillin-clavulanate, which is new to the Canadian market. The majority of patients were successfully de-escalated from piperacillin-tazobactam or a carbapenem for respiratory infections or skin and soft tissue infections. Intravenous amoxicillin-clavulanate provides a good alternative in an era of rising *Pseudomonas aeruginosa* resistance.

Intravenous (IV) amoxicillin-clavulanate has been used in Europe for years with success.^1,2^ This product has been available in Canada since 2020, and on our hospital formulary since 2022. Historically, ceftriaxone is used empirically for inpatients with community-acquired infections, while those with hospital-acquired infections would receive piperacillin-tazobactam.

The prevalence of multidrug resistance *Pseudomonas aeruginosa* is increasing and varies from 15% to 30 worldwide.^3^ In our health authority, the prevalence of *Pseudomonas aeruginosa* for hospital-acquired pneumonia is 15%. Multiple studies showed that exposures to piperacillin-tazobactam and carbapenems were associated with increased risk of multidrug resistant *P. aeruginosa*.^4,5^ Amoxicillin-clavulanate does not have activity against *P. aeruginosa* and is narrower in spectrum than piperacillin-tazobactam^6^; therefore, its use in selected cases may decrease antibiotic resistance.

The objectives of our study are: (1) describe the prescribing pattern of IV amoxicillin-clavulanate since being added to hospital formulary; (2) evaluate whether IV amoxicillin-clavulanate is used appropriately; (3) identify where IV amoxicillin-clavulanate is used to de-escalate from other broad-spectrum antibiotics.

## Methods

This retrospective case series took place at a 447-bed tertiary hospital and a 178-bed community hospital in British Columbia, Canada from October 2022 to April 2023. An antimicrobial stewardship (AMS) pharmacist and infectious diseases (ID)/AMS physician presented to hospitalists, surgeons, and other medical staff at both sites when IV amoxicillin-clavulanate was added to the formulary. Both hospitals had pre-existing AMS prospective audit and feedback, recommendations for de-escalation to IV amoxicillin-clavulanate were incorporated into daily practice following formulary addition.

All admitted adult patients on IV amoxicillin-clavulanate were included. We used AMS software (Lumed, Sherbrooke, Canada) to identify patients who received IV amoxicillin-clavulanate, and collected demographic and clinical data via the electronic medical record. Primary outcomes included appropriateness of indication and duration of IV amoxicillin-clavulanate and clinical success. Appropriate indications for IV amoxicillin-clavulanate were based on provincial guidelines (Supplemental Table 1). Transition to oral therapy was assessed as part of appropriateness of indication on day 3 of IV amoxicillin-clavulanate. Appropriate duration of therapy was based on locally approved guidelines. Clinical success was defined as not requiring re-escalation to piperacillin-tazobactam or a carbapenem during hospitalization within 30 days of IV amoxicillin-clavulanate. Secondary outcomes included readmission rate, reason(s) for readmission, and mortality rate within 30 days of stopping IV amoxicillin-clavulanate. Patients who received less than 24 hours of IV amoxicillin-clavulanate were excluded from evaluation of clinical success and secondary outcomes. Only the first episode of IV amoxicillin-clavulanate use was evaluated for primary and secondary outcomes. An AMS pharmacist reviewed all cases, with verification by an ID/AMS physician for cases with ambiguous outcomes. Descriptive statistics were used for analysis. A letter of exemption from the institutional research ethics board was obtained.

## Results

One hundred and thirteen charts were identified. Two patients were excluded as they did not receive IV amoxicillin-clavulanate per the medication administration record. Patient characteristics, indications, type of prescriber, duration, and reasons for choosing IV amoxicillin-clavulanate are summarized in Table 1. Respiratory tract (complex parapneumonic effusions and empyema), and skin or soft tissue infections (diabetic foot infections and post-operative infections) were the most common indications. IV amoxicillin-clavulanate was used for de-escalation from piperacillin-tazobactam or a carbapenem in 59% of cases.

**Table 1.** Summary of patient demographics and prescribing pattern of intravenous (IV) amoxicillin-clavulanate

**Demographics (*n* = 111)**	
Age (years), mean	68
Male, *n* (%)	70 (63)
**Indications, *n* ^a^**	
Head and neck infections	6
Respiratory tract infections	36
Intra-abdominal infections	13
Urinary tract infections	16
Skin and soft tissue or diabetic foot infections	25
Bone and joint infections	11
Others	7 (eg, fever of unknown origin)
Empiric treatment, *n* (%)	68 (61)
Targeted treatment, *n* (%)	43 (39)
Polymicrobial	17
*E. coli*	10
*Enterococcus* species	8
Methicillin susceptible *S. aureus* only	6
IV amoxicillin-clavulanate used to de-escalate piperacillin-tazobactam or carbapenem, *n* (%)	66 (59.4)
Prescribers can only use IV amoxicillin-clavulanate (ie, ceftriaxone +/– metronidazole is not an option), *n* (%)	34 (30.6)
**Documented reason(s) for choosing IV amoxicillin-clavulanate, n**	
Allergic to ceftriaxone	4
Culture only susceptible to IV amoxicillin-clavulanate	18
Patients already tried ceftriaxone prior to IV amoxicillin-clavulanate	18
Planned for direct transition to oral amoxicillin-clavulanate	28
**Duration of IV amoxicillin-clavulanate, days**	
Mean	6.5
Median	5
**Prescriber of IV amoxicillin-clavulanate**	
Hospitalist	37
Infectious diseases	45
Respirologists	9
Internal medicine	6
Surgeons	9
IV amoxicillin-clavulanate use due to AMS recommendations, n (%)	13 (11.7)

a Does not add up to 111, as patient can have > 1 infection

Primary and secondary outcomes are summarized in Table 2. IV amoxicillin-clavulanate was prescribed appropriately in 95% of cases, of which 12% were based on AMS recommendations. IV amoxicillin-clavulanate was the only option in one-third of the cases (eg culture grew *Enterococcus* species), whereas ceftriaxone with or without metronidazole could have been used in the remaining cases. The main reason for choosing IV amoxicillin-clavulanate in those cases was to facilitate subsequent direct oral transition. ID physicians and hospitalists were the main prescribers. Reasons why transition to oral amoxicillin-clavulanate was not feasible are shown in Table 2.

**Table 2.** Summary of primary and secondary outcomes

**Primary Outcomes,** ***n*** **(%)**	
Appropriateness outcomes (*n* = 111)	
Appropriateness of indication	106 (95.5)
Appropriateness of duration	105 (94.6)
Clinical outcomes (*n* = 97)	
Clinical success	70 (72.2)
Subsequent transition to oral amoxicillin-clavulanate	54 (55.7)
Reason(s) for inability to transition to oral amoxicillin-clavulanate on day 3 of IV amoxicillin-clavulanate (*n* = 55)^a^	
Patient is not improving clinically	29
Inability to swallow	9
Pending specialist reassessment	7
Pending further workup of infection(s) or imaging	7
Patient non-compliant with oral medication	2
Nonfunctioning GI tract	1
Other	4
Reason(s) for re-escalation to piperacillin-tazobactam or a carbapenem (n = 27)	
Patients deteriorated clinically for the same indication	18 (66.7)
Inadequate source control	5 (18.5)
Grew subsequent resistant organisms	3 (11.1)
A different infection developed	1 (3.7)
**Secondary outcomes (** ***n*** **= 97),** ***n*** **(%)**	
Hospital readmission	15 (15.5)
Related to the same infection	4 (26.7)
Readmitted for other reasons	11 (73.3)
Mortality	18 (18.6)
Comfort care/end of life	12 (66.7)

a Does not add up to 55, as patient can have > 1 reason

Clinical success was reviewed in 97 patients, 13 patients received less than 1 day of therapy and were excluded. One patient was lost to follow-up. Clinical success was achieved in 72% of patients, and IV amoxicillin-clavulanate was well tolerated. Clinical deterioration while on treatment was the main reason for re-escalation to other broad-spectrum antibiotics.

## Discussion

There is a scarcity of literature to describe the use of IV amoxicillin-clavulanate in Canada. A pilot study in Alberta evaluated IV amoxicillin-clavulanate as an alternative to piperacillin-tazobactam for general surgery patients. However, the uptake by surgeons was low, which the authors attributed to the unfamiliarity of this new product.^7^ In contrast, we educated a broad range of prescribers (eg, hospitalists, respirologists, general surgeons) when IV amoxicillin-clavulanate was added to formulary, plus ongoing education via prospective audit and feedback.

IV amoxicillin-clavulanate was used for de-escalation from piperacillin-tazobactam in 59% of patients. One can argue that ceftriaxone with or without metronidazole may also be used, but IV amoxicillin-clavulanate was the only choice in one-third of these patients due to culture susceptibility, drug allergy, etc. A number of these patients were also thought to have failed ceftriaxone prior to escalation to piperacillin-tazobactam.

Amoxicillin-clavulanate may have some preferable characteristics compared to ceftriaxone. Third-generation cephalosporins are more strongly associated with healthcare-associated *Clostridioides difficile* infection than beta-lactam-*β*-lactamase inhibitor combinations.^8^ Cephalosporins also have a stronger association than beta-lactam-*β*-lactamase inhibitor combinations with acquiring colonization with extended-spectrum *β*-lactamase-producing Gram-negative bacilli.^9^

Another advantage of IV amoxicillin-clavulanate is that it allows for direct oral transition. The median duration of IV amoxicillin-clavulanate was 5 days. Most patients requiring prolonged therapy (> 7 days) were reviewed by specialists (e.g., ID physicians, respirologists). A number of patients had empyema or complex parapneumonic effusions; these findings were similar to a study by Artoisenet et al.^10^ ID specialists prescribed IV amoxicillin-clavulanate in 40% of cases. Their patients typically had complex skin and soft tissue infections, diabetic foot infections, or bone and joint infections; once deemed not to have risk factors for *P. aeruginosa* or other resistant organisms, they were de-escalated to IV amoxicillin-clavulanate.

Clinical success was achieved in 72% of our patients. The reasons for broadening therapy were multifactorial including inadequate source control, patient risk factors, and prolonged hospitalization leading to growth of multidrug resistant organisms. Fifteen percent of patients were readmitted within 30 days of IV amoxicillin-clavulanate use; the majority were for reasons unrelated to the original infection. Mortality rate was 18%, mainly from cancer-related complications.

One strength of this study is inclusion of both a tertiary hospital and a smaller community hospital to compare their prescribing patterns of IV amoxicillin-clavulanate. We noted that all the inappropriate duration happened at the community hospital; this is possibly due to fewer ID physician and AMS pharmacist present at that site. A limitation is missing information for some patients due to the retrospective nature of this study. We did not evaluate the total days of piperacillin-tazobactam potentially saved by switching to IV amoxicillin-clavulanate. Since the cost of both IV antibiotics is similar, we do not expect substantial drug cost savings with the switch.

In an era of rising antibiotic resistance, IV amoxicillin-clavulanate provides a good alternative for patients who do not require piperacillin-tazobactam. Our study describes those who would benefit from this regimen: empirically for patients with community-acquired respiratory infections (eg empyema), complicated skin and soft tissue infections (eg diabetic foot infections), or when polymicrobial coverage is needed (eg intra-abdominal infections).

## Supporting information

Wong et al. supplementary material Wong et al. supplementary material

## Supplementary material

For supplementary material accompanying this paper visit http://doi.org/10.1017/ash.2024.18. click here to view supplementary material
