# Supplementary material for: Evaluation of intravenous amoxicillin-clavulanate use in two Canadian hospitals
Source: Antimicrob Steward Healthc Epidemiol. 2024 Feb 14;4(1):e24. doi: 10.1017/ash.2024.18 (PMC10897708; doi:10.1017/ash.2024.18)
Supplement: Wong et al. supplementary material [file S2732494X24000184sup001.docx]

**Supplemental Table 1: Appropriate indications for IV amoxicillin-clavulanate based on provincial guidelines**

| **Indications** | **Rationale for using IV amoxicillin-clavulanate and examples** |
| --- | --- |
| Head and neck infections | Severe odontogenic infections in patients unable to take oral medications. |
| Pneumonia (community-acquired, including aspiration pneumonia) | Ceftriaxone (+ metronidazole if anaerobic coverage required) should be used preferentially. However, IV amoxicillin-clavulanate would be an acceptable option. |
| Intra-abdominal infections | For polymicrobial infections not covered by ceftriaxone + metronidazole (e.g., culture showed *Enterococcus faecalis*). |
| Urinary tract infections | For patients who cannot tolerate oral therapy, and urine culture not susceptible to other narrower spectrum antibiotic(s). |
| Polymicrobial skin and soft tissue infections | Human and animal bite infections  Diabetic foot infections (when there are no risk factors for *Pseudomonas aeruginosa*). |
| Bone and joint infections | Ceftriaxone (+ metronidazole if anaerobic coverage required) should be used preferentially. However, IV amoxicillin-clavulanate would be an acceptable option when there are no risk factors for *Pseudomonas aeruginosa.* |
